# Enhancing the volume and the optical quality of hen egg-white lysozyme crystals by coupling the salt concentration gradient crystallization method with a magnetic field

**DOI:** 10.1107/S0021889812036060

**Published:** 2012-09-13

**Authors:** Elena Magay, Sang Jin Cho, Shin Ae Kim

**Affiliations:** a Neutron Science Division, Basic Science and Technology Department, Korea Atomic Energy Research Institute, 989-111 Daedeok-daero, Yuseong-gu, Daejeon, 305-353, Republic of Korea

**Keywords:** lysozyme, crystallization, magnetic fields, optical perfection

## Abstract

The effect of coupling the salt concentration gradient crystallization method with the use of the paramagnetic salt MnCl_2_ and a magnetic field is reported. The use of a simple magnetic device is proposed to have a significant effect on hen egg-white lysozyme crystal growth. Large single crystals greater than 10 mm^3^ in volume with optical perfection were consistently obtained in this study.

## Abstract 2

The effect of coupling the salt concentration gradient crystallization method with the use of the paramagnetic salt MnCl_2_ and a magnetic field is reported. The use of a simple magnetic device is shown to have a significant effect on hen egg-white lysozyme crystal growth.

## 1. Introduction

Neutron diffraction is a powerful technique for the location of proton and/or deuteron positions and thereby provides a more complete picture of atomic structures. However, because of the inherent low flux of neutron sources (some many orders of magnitude less intense than X-ray sources), large crystal volumes are required in order to gain sufficiently high reflection intensities. Even the most intense neutron sources still require protein crystal volumes to be of the order of 1 mm^3^ (or ∼0.1–0.2 mm^3^ if perdeuterated) (Blakeley *et al.*, 2008 ▶; Blakeley, 2009 ▶). As such, of the 83 106 structures deposited in the Protein Data Bank (Berman *et al.*, 2000 ▶) (as of 17 July 2012), only 57 structures have been determined using neutrons (neutron diffraction and hybrid experimental methods). Therefore, in order for the structural biology community to fully benefit from neutron diffraction experiments, the development of crystallization protocols for the routine growth of large protein crystals needs to be addressed.

Hen egg-white lysozyme (HEWL) was chosen for this study because of its commercial availability and its high propensity to crystallize. To routinely supply large crystals of HEWL for testing of a neutron single-crystal diffractometer, we became interested in the crystallization of proteins in the presence of a magnetic field and paramagnetic salts, as this had been shown to result in improvements in crystal volume and quality (Katsuki *et al.*, 2006 ▶; Gavira & Garcia-Ruiz, 2009 ▶).

Here we report the effect of coupling the salt concentration gradient crystallization method with the use of a magnetic field on the volume and optical quality of HEWL crystals.

## 2. Materials and methods

**Figure 1:**
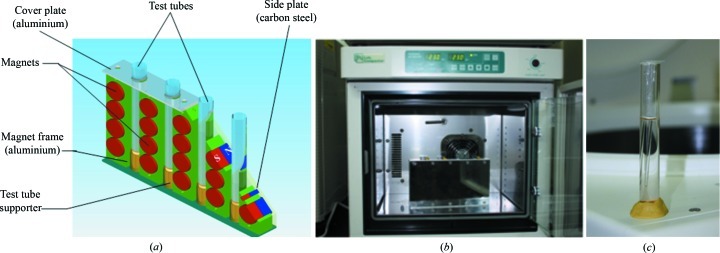
The magnetic device. (*a*) Disassembled position (schematic illustration). (*b*) Assembled position (the magnetic device with the test tubes is placed into an incubator). (*c*) Capillaries (the ‘crystal hanger’) are inserted into the tube.

The crystallization of HEWL (Sigma, L6876) was carried out in a concentration gradient of MnCl_2_. A magnetic device was used to generate a homogeneous 0.3 T magnetic field (Figs. 1 ▶ *a* and 1 ▶ *b*). This device consists of a simply constructed cassette in which small round magnets are compactly packed into an aluminium frame. The protein solutions were prepared from lyophilized powder dissolved in a 50 m*M* Na acetate buffer; the concentrations of protein (30, 40, 50, 60 mg ml^−1^) and pH values (5.0, 5.5, 6.0) were both varied. All solutions were filtered through a 0.20 µm Sartorius filter. The MnCl_2_ powder (1 g) was placed at the bottom of vertically held tubes, 100 mm in length, and an aqueous solution of HEWL was carefully applied on top of the salt. Glass capillaries (the ‘crystal hanger’; Fig. 1 ▶ *c*) were used to suppress nucleation on the walls of the tubes (Magay & Yoon, 2011 ▶). After protein solutions had been prepared, the tubes were immediately inserted into the magnetic device, to apply a magnetic field during the protein crystal growth, and the whole assembly was placed into an incubator. The MnCl_2_ powder dissolved within a few hours and started to diffuse upwards. The tubes kept out of the magnetic device were denoted as the control case. The experiments were performed at 291 and 296 K. Ten crystals of each cohort, with and without magnetic field, from identical growth areas were mounted into quartz capillaries and subjected to comparative measurement of crystal volume. The measurement was carried out using a Dino-Lite and Dino-Eye digital microscope.

## 3. Results

**Figure 2:**
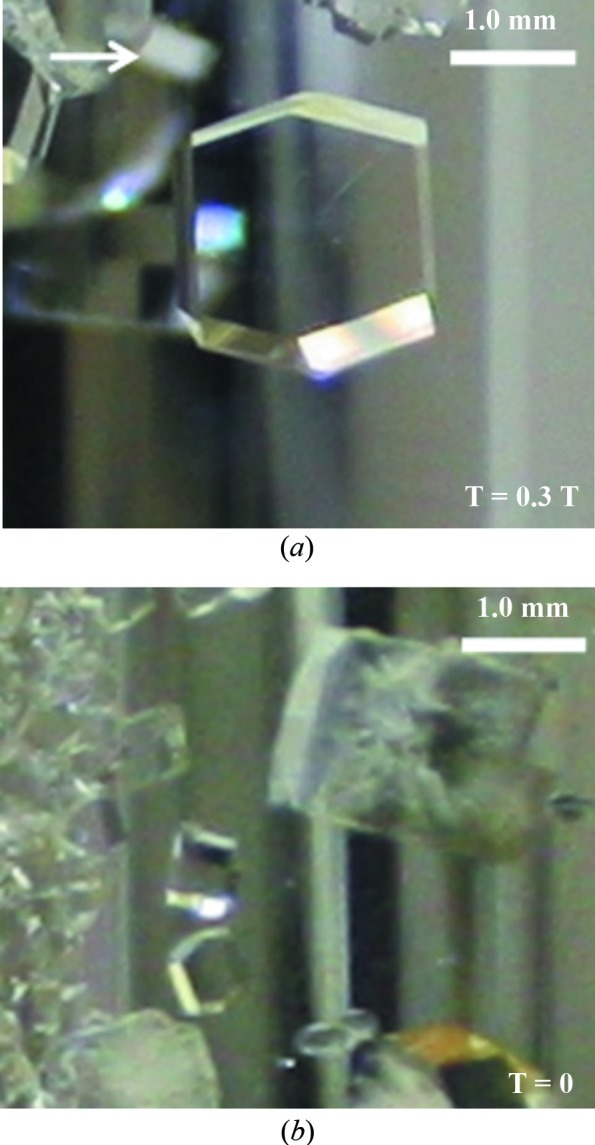
The effect of the magnetic field on the quality of crystals. Photographs were taken 14 d after the start of the experiment. The direction of the magnetic field is shown by the arrow. (*a*) A crystal grown in the presence of a magnetic field has perfect transparency. (*b*) A crystal grown in the absence of a magnetic field has many micro-cracks.

**Figure 3:**
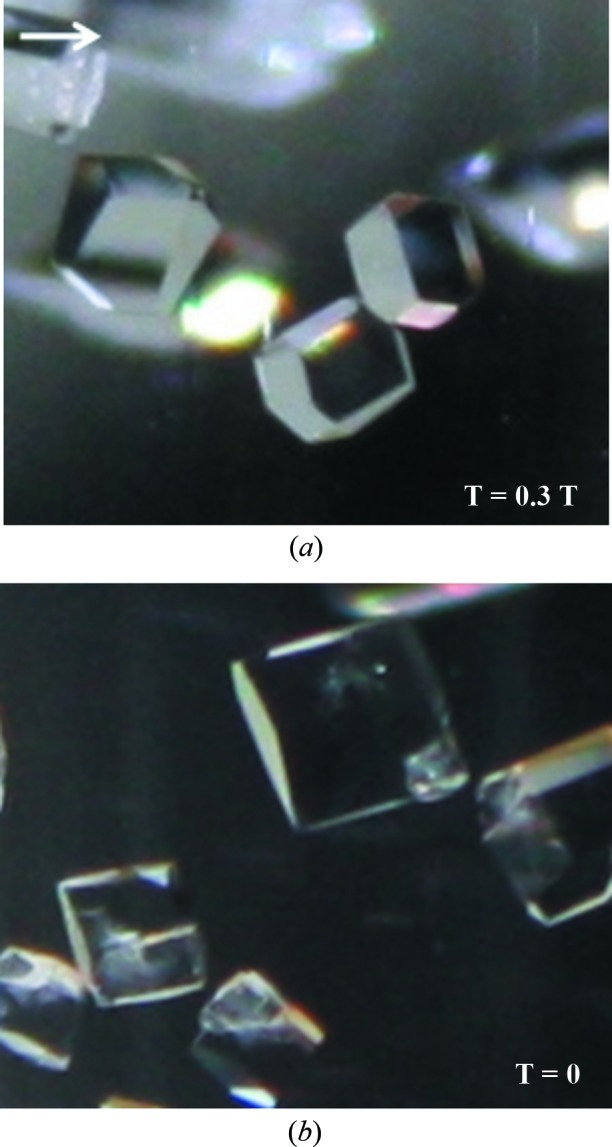
The effect of the magnetic field on the morphology of crystals. Photographs were taken 7 d after the start of the experiment. The direction of the magnetic field is shown by the arrow. (*a*) Crystals have a ‘hexagonal’ shape in the presence of a magnetic field. (*b*) Crystals have a ‘square’ shape in the absence of a magnetic field.

In both control and test cases the crystals hardly achieved 1 mm in length at 291 K, irrespective of the pH level. A significant improvement in crystal volume was detected at 296 K. The crystals obtained after three weeks at a height of 20–40 mm were measured. The crystals were larger near to the center of the growth area and smaller close to the edges. Therefore, the crystals had a large spread of volumes (supplementary data^1^). The average volume of single crystals grown with a magnetic field was measured to be greater than 10 mm^3^ (average 10.6 mm^3^; standard deviation 6.0 mm^3^), and the average volume of crystals grown without a magnetic field was registered as slightly more than 3 mm^3^ (average 3.1 mm^3^; standard deviation 2.8 mm^3^). The ‘best’ yield of crystals was obtained at 50 mg ml^−1^ with 50 m*M* Na acetate at pH 6.0 at 296 K in the presence of a magnetic field (Fig. 2 ▶ *a*). We observed that the crystals were optically perfect in both control and test cases in their early stage of growth (2–5 d from the setting of the experiment). However, the crystals grown in the presence of a magnetic field were finally much larger and had perfect transparency, whereas the crystals grown in the absence of a magnetic field had micro-cracks (Fig. 2 ▶ *b*). Notably, the crystals had a ‘hexagonal’ shape in the presence of a magnetic field and a ‘square’ shape in the absence of a magnetic field (Fig. 3 ▶). The protein concentration did not contribute in any significant manner. Experiments that were carried out at a higher temperature (301 K) and pH level (6.5) showed an irregular formation of crystals. Therefore, these experiments were discontinued. The coupling of the salt concentration gradient method with the use of the paramagnetic salt MnCl_2_ and a magnetic field has proved to significantly improve the volume and optical quality of HEWL crystals.

## 4. Discussion

Divalent paramagnetic cations show a tendency to grow larger crystals than ordinary diamagnetic salts, and Mn^2+^ ions can make crystals that look optically better in a magnetic field (Yin *et al.*, 2003 ▶). The ‘crystal hanger’ acts as an ‘artificial’ nucleation site that induces crystallization around the capillaries, leading to the suppression of nucleation on the walls of the tube and resulting in large single crystals (Magay & Yoon, 2011 ▶). While the ‘crystal hanger’ regulates excessive nucleation, the magnetic field controls the optical quality and shapes of the crystals. The preference of crystals to be crystallized in a certain shape is their response to the magnetic field (Tanimoto *et al.*, 2002 ▶). The technique developed here provides a consistent method for obtaining large and optically clear single crystals of HEWL. The proposed magnetic device does not require a cumbersome water bath and heat insulator, in contrast to general magnetic field generators (Yin *et al.*, 2003 ▶), and can test four samples, whereas a previously described superconducting magnetic device tests only two samples (Yin *et al.*, 2009 ▶). The results clearly demonstrate the efficiency of coupling the salt concentration gradient method with the paramagnetic salt MnCl_2_ as a crystallizing agent and a magnetic field. Hence, the demand for crystals of HEWL with volumes greater than 1 mm^3^ for neutron experiments can be achieved routinely.

We intend to apply our method to vapor diffusion and micro-batch techniques to extend the limits on crystal growth of proteins that are only available in small supply, unlike lysozyme which can be purchased by the gram. Currently, magnetic devices suitable for such experiments are under construction.

## Supplementary Material

Supplementary material file. DOI: 10.1107/S0021889812036060/he5553sup1.pdf
Crystal volume measurement

## Footnotes

1 Supplementary data for this paper are available from the IUCr electronic archives (Reference: HE5553). Services for accessing these data are described at the back of the journal.
