# Supplementary material for: Enhancing the volume and the optical quality of hen egg-white lysozyme crystals by coupling the salt concentration gradient crystallization method with a magnetic field
Source: J Appl Crystallogr. 2012 Sep 13;45(Pt 5):1066–8. doi: 10.1107/S0021889812036060 (PMC3445808; doi:10.1107/S0021889812036060)
Supplement: Supplementary file 1 [file j-45-01066-sup1.pdf]

Crystal volume measurement

T=0.3 #	a - length	b - width	c- height	volume (mm3)
1	2.4	2.2	1.2	6.4
2	3.4	3.1	1.9	19.8
3	3.4	2.6	2.3	19.3
4	2.3	2.1	1.6	7.6
5	2.3	2.2	1.1	5.3
6	3.0	2.9	2.1	18.5
7	2.5	2.2	1.4	7.9
8	2.8	2.4	1.3	8.6
9	2.0	2.0	1.6	6.4
10	2.2	2.0	1.5	6.4
			average	10.6
			stdev	6.0

T=0<br>#	a - length	b - width	c- height	volume (mm3)
1.0	2.2	2.0	1.6	6.9
2.0	2.1	2.0	2.0	8.8
3.0	1.8	1.7	1.4	4.3
4.0	1.0	1.0	0.4	0.4
5.0	2.0	1.5	0.9	2.7
6.0	1.7	1.8	0.9	2.8
7.0	1.4	0.7	0.7	0.7
8.0	1.2	1.0	0.9	1.0
9.0	1.4	1.3	1.0	1.7
10.0	1.4	1.4	1.0	1.9
			average	3.1
			stdev	2.8
